# Candidate gene associations with mood disorder, cognitive vulnerability, and fronto-limbic volumes

**DOI:** 10.1002/brb3.226

**Published:** 2014-03-18

**Authors:** Thomas W Frazier, Eric A Youngstrom, Brian A Frankel, Giovana B Zunta-Soares, Marsal Sanches, Michael Escamilla, David A Nielsen, Jair C Soares

**Affiliations:** 1 Centers for Autism and Pediatric Behavioral Health, Cleveland ClinicCleveland, Ohio; 2 Departments of Psychology and Psychiatry, University of North Carolina at Chapel HillChapel Hill, North Carolina; 3 Menninger Department of Psychiatry and Behavioral Sciences, Baylor College of Medicine and Michael E. DeBakey VA Medical CenterHouston, Texas; 4 Department of Psychiatry and Behavioral Sciences, UT Center of Excellence on Mood Disorders, University of Texas Medical School at HoustonHouston, Texas; 5 Center of Excellence in Neurosciences, Texas Tech University Health Science CenterEl Paso, Texas

**Keywords:** *ANK3*, *BDNF*, bipolar disorder, *CACNA1C*, candidate gene, *DGKH*, major depression, mediation, mood disorder, structural neuroimaging

## Abstract

**Background:**

Four of the most consistently replicated variants associated with mood disorder occur in genes important for synaptic function: *ANK3* (rs10994336), *BDNF* (rs6265), *CACNA1C* (rs1006737), and *DGKH* (rs1170191).

**Aims:**

The present study examined associations between these candidates, mood disorder diagnoses, cognition, and fronto-limbic regions implicated in affect regulation.

**Methods and materials:**

Participants included 128 individuals with bipolar disorder (33% male, Mean age = 38.5), 48 with major depressive disorder (29% male, Mean age = 40.4), and 149 healthy controls (35% male, Mean age = 36.5). Genotypes were determined by 5′-fluorogenic exonuclease assays (TaqMan®). Fronto-limbic volumes were obtained from high resolution brain images using Freesurfer. Chi-square analyses, bivariate correlations, and mediational models examined relationships between genetic variants, mood diagnoses, cognitive measures, and brain volumes.

**Results:**

Carriers of the minor *BDNF* and *ANK3* alleles showed nonsignificant trends toward protective association in controls relative to mood disorder patients (*P* = 0.047). *CACNA1C* minor allele carriers had larger bilateral caudate, insula, globus pallidus, frontal pole, and nucleus accumbens volumes (smallest *r* = 0.13, *P* = 0.043), and increased IQ (*r* = 0.18, *P* < 0.001). *CACNA1C* associations with brain volumes and IQ were independent; larger fronto-limbic volumes did not mediate increased IQ. Other candidate variants were not significantly associated with diagnoses, cognition, or fronto-limbic volumes.

**Discussion and conclusions:**

*CACNA1C* may be associated with biological systems altered in mood disorder. Increases in fronto-limbic volumes and cognitive ability associated with *CACNA1C* minor allele genotypes are congruent with findings in healthy samples and may be a marker for increased risk for neuropsychiatric phenotypes. Even larger multimodal studies are needed to quantify the magnitude and specificity of genetic-imaging-cognition-symptom relationships.

## Introduction

Understanding relationships between candidate genes and mood disorder is crucial for advancing toward molecular-based treatment approaches. Several candidate genes have been identified for mood disorders (Kupfer et al. 2012; Sullivan et al. 2012), with the strongest statistical signals for bipolar disorder (Lohoff et al. 2005; Baum et al. 2008; Ferreira et al. 2008). However, genome-wide association studies of common variants suggest that only a small proportion of the disease is accounted for by accumulation of these variants (Cichon et al. 2009). The modest fraction of phenotypic variance explained is likely a function of the heterogeneity of mood disorders, even within specific categories (Kupfer et al. 2012).

An important intermediate step is evaluation of relationships between candidate genes and structural brain changes or cognitive processes implicated in mood disorders (Gottesman and Gould 2003; Drevets et al. 2008). Structural and functional fronto-limbic brain abnormalities have been implicated in mood disorders (Drevets et al. 2008), most prominently bipolar disorder (Price and Drevets 2010). Additionally, a broad range of cognitive deficits have been observed in mood disorder. Numerous studies have identified memory impairments and impulsivity as likely trait characteristics in bipolar disorder (Robinson et al. 2006; Joseph et al. 2008; Bora et al. 2009; Kurtz and Gerraty 2009) and, to a lesser extent, major depression (Snyder 2013). These endophenotypes may be more closely related to genetic variation (Gottesman and Gould 2003) and provide a window into specific vulnerabilities that increase the probability of mood disorder evolution.

A growing number of studies have evaluated the relationships between candidate genes and cognitive processes or brain volumes in healthy and mood disordered samples (Bigos et al. 2010; Krug et al. 2010*;* Thimm et al. 2011; Frodl et al. 2012; Radua et al. 2012). For example, Frodl et al. (2012) identified associations of genes important for glucocorticoid and immune function with hippocampal volume in patients with major depressive disorder. Previous data from our group has found altered anterior cingulate volumes in individuals with bipolar disorder who carried the *BDNF* minor allele (Matsuo et al. 2009). A single nucleotide polymorphism (SNP) rs1006737 in *CACNA1C,* implicated in bipolar disorder (Ferreira et al. 2008) and other neuropsychiatric disorders (Gargus 2006, 2009), has been found to increase brain volumes, particularly grey matter volumes (Kempton et al. 2009), and impair appropriate functioning of fronto-temporal circuits (Wang et al. 2011) important for emotional processing (Radua et al. 2012). Similarly, variations in *ANK3* and *DGKH*, also implicated in bipolar disorder (Baum et al. 2008) and other neuropsychiatric disorders (Weber et al. 2011), have been associated with altered brain structure and function (Hatzimanolis et al. 2012; Linke et al. 2012; Whalley et al. 2012). Taken together, these data support a model where genes important for ongoing neural plasticity and immune system functioning influence cognitive and structural brain endophenotypes representing key nodes of mood disorder vulnerability.

The present study evaluated four strong candidate polymorphisms from genes important for neurotransmission and plasticity - *ANK3* (rs10994336), *BDNF* (rs6265), *CACNA1C* (rs1006737), and *DGKH* (rs1170191). The functionality of the intronic variants in ANK3, CACNA1C, and DGKH have not been demonstrated. However, it was reported that CACNA1C rs1006737 AA genotype subjects have greater mRNA expression in the dorsolateral prefrontal cortex than subjects with GG or GA genotypes (Bigos et al. 2010). Additionally, each of these candidate polymorphisms is believed to have functional relevance to neuropsychiatric disorders, including mood disorder: ANK3 is thought to influence the function of voltage-gated sodium channels, BDNF regulates neuronal growth and participates in plasticity of neurons throughout the lifespan, CACNA1C is the alpha 1C subunit of the L-type voltage-gated calcium channel, and DGKH participates in the lithium-sensitive phosphatidyl inositol pathway. Therefore, the primary aim of this study was to explore associations between these candidate genes, cognitive processes associated with mood disorder, and mood symptoms and diagnoses. Associations were investigated in a well-characterized sample of adults with bipolar disorder, major depressive disorder, or healthy controls. Candidate polymorphisms were expected to show only modest associations with mood disorder symptoms and diagnoses. However, genetic variation was expected to be significantly associated with individual differences in cognitive processing (global ability, impulsivity, memory) and fronto-limbic volumes. Fronto-limbic volumes were expected to mediate the relationships between genetic variation and cognitive vulnerability to mood disorder.

## Materials and Methods

### Participants

The present sample represents a subgroup of individuals accrued through multiple diagnostic clinics and recruited into overlapping NIMH-funded research studies evaluating neuroimaging findings in adults with mood disorders at the University of Texas Health Science Center at San Antonio. In these studies, adult participants were recruited using advertisements broadcast on the radio and flyers placed in the community and at hospitals and clinics in the South Texas Medical Center area. Age, gender, handedness, and race/ethnicity (coded as white/non-Hispanic and other race/ethnicity) were obtained via clinical interview. Participants received a physical examination and laboratory tests to rule out physical illnesses and substance use. Any participant with endocrinological disease, head trauma, neurological disease, family history of hereditary neurological disorder, or a medical condition such as hypertension, diabetes, active liver disease, kidney problems, respiratory problems, or current alcohol /drug abuse dependence was excluded. Left handed and ambidextrous participants were excluded from this sample to reduce heterogeneity of neuroimaging.

The Institutional Review Board of the University of the Texas Health Science Center at Houston and Baylor College of Medicine approved this study. Written informed consent was obtained from all the participants after a complete description of the study was provided.

### Procedures

#### Diagnostic and symptom assessment

Participants were evaluated for DSM-IV-TR Axis I disorders using the Structured Clinical Interview for DSM-IV (SCID) Axis I disorders, research version, patient edition (First et al. 2002). A senior psychiatrist (JCS) reviewed all clinical information, including history of medical and neurological conditions, and confirmed that all subjects met DSM-IV-TR diagnostic criteria for bipolar disorder (BD) or for Major Depressive Disorder. Clinical symptom ratings were completed using the Hamilton Rating Scale for Depression (HAM-D; Hamilton 1960) and the Young Mania Rating Scale (YMRS; Young et al. 1978). Healthy comparison participants were eligible if they did not have any DSM-IV axis-I disorder, as assessed by trained psychiatrists using the SCID-I nonpatient version, any history of alcohol/substance abuse or dependence, and history of any psychiatric or neurological disorders in any of their first-degree relatives. Exclusion criteria for all participants were as follows: age less than 18 years, current serious medical conditions, history of head trauma, organic mental disorders, or neurological disorders. An additional exclusion criterion for bipolar disorder patients was history of alcohol/substance abuse or dependence within the 6 months preceding study entry.

#### Cognitive measures

Verbal ability was estimated via the standard score from the Wechsler Test of Adult Reading (Wechsler 2001). Nonverbal ability was estimated using the Test of Nonverbal Intelligence (Brown et al. 1997). Full scale intelligence quotient (IQ) was estimated by averaging scores on these measures. Long-term verbal memory was evaluated using the total learning score from trials 1–5 of the California Verbal Learning Test (CVLT; Delis et al. 1987) Sustained attention and impulsive responding were evaluated using total hits, mean reaction time, and false alarms from the Identical Pairs-Continuous Performance Test (IPCPT; Cornblatt and Malhotra 2001).

#### Structural brain volumes

High resolution 3D brain images were acquired on a Philips 1.5 T MR system (Philips Medical System, Andover, MA). Images were collected by means of an axial three-dimensional, T1-weighted, fast field echo sequence (field of view 256 mm; view matrix 256 · 256; repetition time 24 ms; echo time 5 ms; flip angle 40 degrees, slice thickness 1 mm). For the present study, volumetric measurements were extracted through a standard procedure using Freesurfer software (Greve and Fischl 2009; Postelnicu et al. 2009; Fischl 2012) version 4.5.0 (http://surfer.nmr.mgh.harvard.edu/). Specifically, the ‘recon-all’ command embedded within Freesurfer was executed for all T_1_-weighted scan data and resulting anatomical volumes used for subsequent statistical analyses.

#### Genotyping

DNA came from blood samples drawn from the study subjects. White blood cells were first separated from plasma, and then the PUREGENE, Gentra Systems, assay was used to isolate the DNA from each subject. Genotypes were determined using a 5′-fluorogenic exonuclease assay (TaqMan®, Applied Biosystems, Foster City, CA). The *ANK3* (rs10994336), *BDNF* (rs6265), *CACNA1C* (rs1006737), and *DGKH* (rs1170191) genotypes were determined using the TaqMan® primer-probe sets (Applied Biosystems) Assay ID C_31344821_10 (rs10994336), C_11592758_10 (rs6265), C_2584015_10 (rs1006737), and C_7448168_10 (rs1170191). PCR amplification was performed using Platinum® quantitative PCR SuperMix-UDG (Invitrogen, Carlsbad, CA) on a GeneAmp® PCR system 9700. Samples were amplified at 50°C for 2 min, 95°C for 10 min, and then 50 cycles of 95°C for 15 s, and 60°C for 1 min. The amplification products were analyzed using an Applied Biosystems Prism® 7900 sequence detection system and SDS 2.2 software (Applied Biosystems). TaqMan® assays were performed in duplicate by an individual unaware of the clinical status of the subjects.

We were unable to obtain any genotype information for the DNA from 31 of the subjects of the 325 subjects in our cohort. Of the remaining 294 subjects, the ANK3 rs10994336 assay had 246 genotypes in concordance between the first and the second assay runs, 21 samples were genotyped in one run only with “undetermined” calls in the other run, and 54 samples failed genotyping in both runs. Three samples produced ANK3 rs10994336 genotypes which were discordant between runs and were excluded from the analyses. The BDNF rs6265 assay had 240 samples in concordance between the first and the second genotype runs, 38 genotypes were determined with information from only one run, and 16 samples failed genotyping in both runs. No samples were discordant for BDNF rs6265 between runs. The CACNA1C rs1006737 assay had 245 samples in concordance between the first and the second runs, 15 calls were made in one run with “undetermined” calls in the other run, and 34 samples failed genotyping in both runs. No samples were discordant for CACNA1C rs1006737 between runs. The ANK3 rs1170191 assay had 214 samples in concordance between the first and the second runs, 21 genotypes were made in only one run, and 55 samples failed genotyping in both runs. Four samples were discordant for ANK3 rs1170191 between runs and were excluded from the analyses. The genotype frequencies for BDNF, CACNA1C, and DGKH were in Hardy–Weinberg equilibrium in the control, bipolar disorder, and major depression groups (*P* > 0.05). The ANK3 genotype frequencies deviated from Hardy–Weinberg equilibrium in all three groups (control, *P* = 0.038; bipolar, *P* = 0.026; and major depression, *P* = 0.015).

### Statistical analysis

#### Power

Statistical power was calculated for the combined sample as bivariate associations were computed in the full sample (across diagnostic groups). The full sample was used based on the growing understanding of within and between group diagnostic heterogeneity and the fact that the primary focus of the study was on genotype-cognition and genotype-brain volume relationships irrespective of diagnosis. The ability to detect significant correlations among measures at different levels of the genotype-phenotype pathway (ex. SNP – brain volume) was estimated to be excellent (0.99) for detecting medium-sized relationships (*r* = 0.30) and very good (0.81) for detecting small to medium relationships (*r* = 0.20), assuming a minimum sample size of *N* = 200 and two-tailed *α* = 0.05. Statistical power remains excellent (>0.87) for detecting medium effect sizes (*r* = 0.30) even at sample sizes as low as 100 – which is smaller than both the bipolar and control subgroups. Power to detect mediation is complex and depends on multiple factors, but is heavily influenced by the ability to detect significance of the indirect effects from the upstream independent variable (ex. SNP) through the mediator (imaging volumes) to the downstream dependent variable (cognitive process or symptom levels; Fritz and MacKinnon 2007). To examine the power to simultaneously detect significance in these indirect paths, a simulation study (*K* = 10,000, *N* = 200, *α* = 0.05, two-tailed) was conducted where the indirect path parameters (equivalent to *β* in regression) were estimated using small and medium effect sizes for both paths (*β*s = 0.20 or 0.39) and the direct effect was specified to be null (*β* = 0.00), representing full mediation. Results of this simulation indicated adequate ability to simultaneously detect smaller indirect effects (*β*s = 0.20; power = 0.63) and excellent ability to detect medium indirect effects (*β*s = 0.39; power > 0.99).

#### Bivariate and multivariate relationships

Bivariate relationships were evaluated using Pearson correlation coefficients for pairs of continuous variables, univariate ANOVA for continuous-ordinal variable combinations, and Pearson chi-square for dichotomous/ordinal pairs. Candidate gene comparisons were analyzed using a dichotomous code comparing major homozygote carriers and minor allele carriers. All analyses were recomputed with race/ethnicity (coded white non-Hispanic, white Hispanic, and other race/ethnicity) as a covariate to ensure that genetic relationships were not confounded by race/ethnicity (Lanktree et al. 2009; Lin et al. 2011; Liu et al. 2012). A false discovery rate correction was applied within each candidate SNP to maintain Type I error rates. Quantile-quantile plots evaluated whether a systematic deviation of bivariate relationships from the null expectation was observed.

Mediational models were computed only for candidate SNPs, brain volumes, cognitive, and symptom/diagnostic variables showing significant bivariate relationships. These models were sequenced to determine whether structural volumes are driving relationships between genotype and cognitive or symptom/diagnostic measures using the Baron and Kenny framework (Baron and Kenny 1986).

For association analyses of minor alleles in the *ANK3*, *BDNF*, *CACNA1C*, and *DGKH* with phenotypes of any mood disorder, bipolar disorder, or major depression, a significant association, after correction for multiple testing, was set at 0.05/12 = 0.0042. False discovery rate corrections were applied within each SNP when examining associations between genotypes and clinical characteristics, cognitive measures, and structural brain volumes to maintain the Type 1 error rate at 0.05 (Benjamini and Hochberg 1995, 2000).

## Results

### Sample characteristics

Table 1 presents sample demographic and clinical characteristics by diagnostic group. Diagnostic groups showed similar age, gender, and race/ethnicity distributions. Education was highest in healthy controls and lowest in bipolar disorder patients. As expected, bipolar disorder patients had higher mania symptom levels and both mood disorder groups had elevated depression levels and worse global functioning. Age of illness onset was slightly lower in bipolar disorder relative to major depression. The majority of patients with bipolar disorder were diagnosed with BP1 followed by BP2. Most patients with major depression had a history of recurrent episodes. Overall and verbal IQ were similar across groups, while healthy controls had slightly higher non-verbal IQ scores relative to both mood disorder groups. Both patients with bipolar and major depression had worse memory performance on CVLT total trials than the control group. On the continuous performance task, patients with bipolar had worse target detection and slower responding relative to healthy controls and patients with major depression. Current medication use and past history of substance use disorder were available for 66% (84 of 128) of patients with bipolar disorder and 83% (40 of 48) of patients with major depression. A large majority of patients with mood disorder had a history of substance use disorder in this sample (44%; 55 of 124). There were no significant differences in the proportions of past history of substance use disorder between patients with bipolar disorder and patients with major depression (χ^2^(1) = 2.09, *P* = 0.148). Patients with bipolar disorder were much more likely to be using psychotropic medication than patients with major depression at the time of the study (34.5% vs. 5.0%; χ^2^(1) = 12.60, *P* < 0.001).

**Table 1:** Sample demographic and clinical characteristics by diagnostic group

	Healthy controls	Bipolar disorder	Major depression	
	Mean (SD)	Mean (SD)	Mean (SD)	F/χ^2^, *P*
N	149	128	48	
Age	36.5 (13.2)	38.5 (12.0)	40.4 (12.3)	*F*(2,322) = 2.07, *P* = 0.127
Male (%)	52 (35.1)	42 (32.8)	14 (29.2)	χ^2^(2) = 0.61, *P* = 0.738
Education	5.5 (1.8)	4.4 (1.6)	4.8 (1.8)	*F*(2,314) = 16.01, *P* < 0.001
Race/ethnicity				
White	71 (47.7)	78 (60.9)	27 (56.3)	χ^2^(4) = 8.04, *P* = 0.090
Hispanic	58 (38.9)	36 (28.2)	19 (39.6)	
Other	20 (13.4)	14 (10.9)	2 (4.2)	
YMRS	0.4 (0.8)	6.4 (7.0)	2.3 (3.0)	*F*(2,315) = 61.98, *P* < 0.001
HAM-D	0.9 (1.4)	13.4 (8.5)	11.6 (9.5)	*F*(2,315) = 137.83, *P* < 0.001
GAF	91.8 (5.3)	63.5 (12.1)	71.5 (16.0)	*F*(2,303) = 244.20, *P* < 0.001
Age of illness onset		18.4 (8.5)	22.1 (9.0)	*F*(1,171) = 6.58, *P* = 0.011
Bipolar diagnoses (%)				
BP 1		96 (75.6)		
BP 2		27 (21.3)		
BP NOS/CYC		4 (3.1)		
Major depression (%)				
Single episode			13 (27.1)	
Recurrent			35 (72.9)	
Full Scale IQ	100.2 (10.2)	97.7 (10.4)	98.6 (9.8)	*F*(2,255) = 1.60, *P* = 0.205
Verbal ability	108.5 (13.5)	108.5 (12.6)	107.5 (14.7)	*F*(2,240) = 0.09, *P* = 0.916
Nonverbal ability	92.3 (10.9)	88.8 (10.4)	89.6 (9.9)	*F*(2,253) = 3.20, *P* = 0.042
CVLT total trials 1–5	52.4 (9.2)	47.4 (10.3)	47.3 (10.7)	*F*(2,256) = 8.45, *P* < 0.001
IPCPT – true positives	41.4 (8.0)	36.6 (9.7)	39.6 (8.4)	*F*(2,252) = 8.06, *P* < 0.001
IPCPT – reaction time	474.5 (50.1)	501.2 (54.9)	484.4 (47.6)	*F*(2,252) = 7.27, *P* = 0.001
IPCPT – false Alarms	3.1 (2.9)	3.9 (3.5)	3.0 (2.7)	*F*(2,252) = 2.33, *P* = 0.100

YMRS, Young Mania Rating Scale, HAM-D, Hamilton Rating Scale for Depression, GAF, Global Assessment of Functioning. BP1, Bipolar 1 Disorder, BP 2, Bipolar 2 Disorder, BP NOS, Bipolar Disorder Not Otherwise Specified, CYC, Cyclothymia. Education N, 317, YMRS N, 318, HAM-D N, 318, GAF N, 306, Age of Illness Onset N, 173.

### Missing data

Imaging data were present for the entire sample. Genotype data were available for the majority of the sample (*ANK3 n* = 268, 83%; *BDNF n* = 281, 87%; *CACNA1C n* = 251, 80%; *DGKH n* = 235, 72%). Missing value analysis indicated that the hypothesis that genotype data were missing completely at random was not rejected (χ^2^(25) = 33.17, *P* = 0.127), implying the influence of missing data was modest.

### Genetic correlates of mood diagnoses and symptoms

Table 2 presents the genotype frequencies by diagnostic group. Patients carrying one or two *BDNF* minor alleles (GA or AA genotypes) showed a nominally significant association with healthy controls, implying a protective effect of this allele for mood disorder. This effect remained significant when adjusting for race/ethnicity (*P* = 0.034). However, when corrected for multiple testing (four different genes tested), this association was no longer significant. *ANK3, CACNA1C,* and *DGKH* genotype groups were not associated with the presence of mood disorder. Table 3 presents relationships between candidate SNPs and clinical characteristics. There were no significant associations between SNPs and mania or depression symptom levels or global psychosocial functioning.

**Table 2:** Genotype frequencies by diagnostic group

		Healthy controls	Bipolar disorder	Major depression	3-Group comparison	Any mood disorder
	Genotype/MAF	*N* (%)	*N* (%)	*N* (%)	χ^2^(*P*)	χ^2^(*P*)
*ANK3*	N	124	101	43	χ^2^(2) = 3.50, *P* = 0.174	χ^2^(1) = 3.40, *P* = 0.065
	GG	85 (68.5)	80 (79.2)	33 (76.7)		
	GA	31 (25.0)	17 (16.8)	7 (16.3)		
	AA	8 (6.5)	4 (4.0)	3 (7.0)		
	MAF	47 (19.0)	25 (12.4)	13 (15.1)		
*BDNF*	N	135	102	44	χ^2^(2) = 3.98, *P* = 0.136	χ^2^(1) = 3.95, *P* = 0.047
	GG	85 (63.0)	75 (73.5)	33 (75.0)		
	GA	41 (30.4)	22 (21.6)	11 (25.0)		
	AA	9 (6.7)	5 (4.9)	0 (0)		
	MAF	59 (21.9)	32 (15.7)	11 (12.5)		
*CACNA1C*	N	123	91	45	χ^2^(2) = 0.31, *P* = 0.858	χ^2^(1) = 0.01, *P* = 0.918
	GG	58 (47.2)	42 (46.2)	23 (51.1)		
	GA	49 (39.8)	38 (41.8)	16 (35.6)		
	AA	16 (13.0)	11 (12.1)	6 (13.3)		
	MAF	81 (32.9)	60 (32.9)	28 (31.0)		
*DGKH*	N	117	81	41	χ^2^(2) = 1.67, *P* = 0.434	χ^2^(1) = 0.25, *P* = 0.614
	AA	79 (67.5)	48 (59.3)	28 (68.3)		
	AC	31 (26.5)	24 (30.4)	10 (25.6)		
	CC	7 (6.0)	7 (8.9)	1 (2.6)		
	MAF	45 (19.2)	38 (23.5)	12 (14.6)		

MAF, minor allele frequency. *ANK3* (rs10994336), *BDNF* (rs6265), *CACNA1C* (rs1006737), *DGKH* (rs1170191). The three-group comparison contrasts major homozygote carriers and minor allele carriers across the three diagnostic groups. The any mood disorder comparison contrasts major homozygote carriers and minor allele carriers with mood disorder groups lumped together (Bipolar Disorder plus Major Depression) versus healthy controls.

**Table 3:** Relationships between genotype and clinical factors. Positive correlations indicate that minor allele carriers are associated with higher scores on clinical factors

	*ANK3* rs10994336	*BDNF* rs6265	*CACNA1C* rs1006737	*DGKH* rs1170191
	*r*	*r*	*R*	*r*
YMRS	−0.09	−0.02	−0.06	0.06
HAM-D	−0.06	−0.08	0.03	0.05
GAF	0.11	0.08	0.03	−0.04
Full Scale IQ	−0.11	0.02	0.18**	−0.07
Verbal ability	−0.16*	0.02	0.20**	0.01
Nonverbal ability	−0.01	0.02	0.15*	−0.13
CVLT Total Trials 1–5	−0.01	0.08	0.06	−0.15*
IPCPT – True positives	0.05	−0.01	−0.04	−0.01
IPCPT – Reaction time	−0.09	−0.08	0.08	0.01
IPCPT – False alarms	0.08	0.03	−0.10	−0.01

* *P* < 0.05,

** *P* < 0.01. Significant relationships for *CACNA1C* rs1006737 survive false discovery rate correction.

### Genetic correlates of cognitive processes and fronto-limbic volumes

Table 3 also presents relationships between the candidate SNPs and cognitive processes related to mood disorder vulnerability. *CACA1C* minor allele carriers (GA and AA genotypes) were associated with higher overall, verbal, and nonverbal IQ scores. *ANK3*, *BDNF*, and *DGKH* genotype groups were not significantly associated with IQ. These relationships remained significant after covarying for race/ethnicity. *DGKH* minor allele carriers (AC and CC genotypes) had reduced verbal learning and memory. However, this did not survive false discovery rate correction. None of the remaining SNPs evaluated were found to be significantly associated with memory or attention/impulsivity measures.

**Figure 1:**
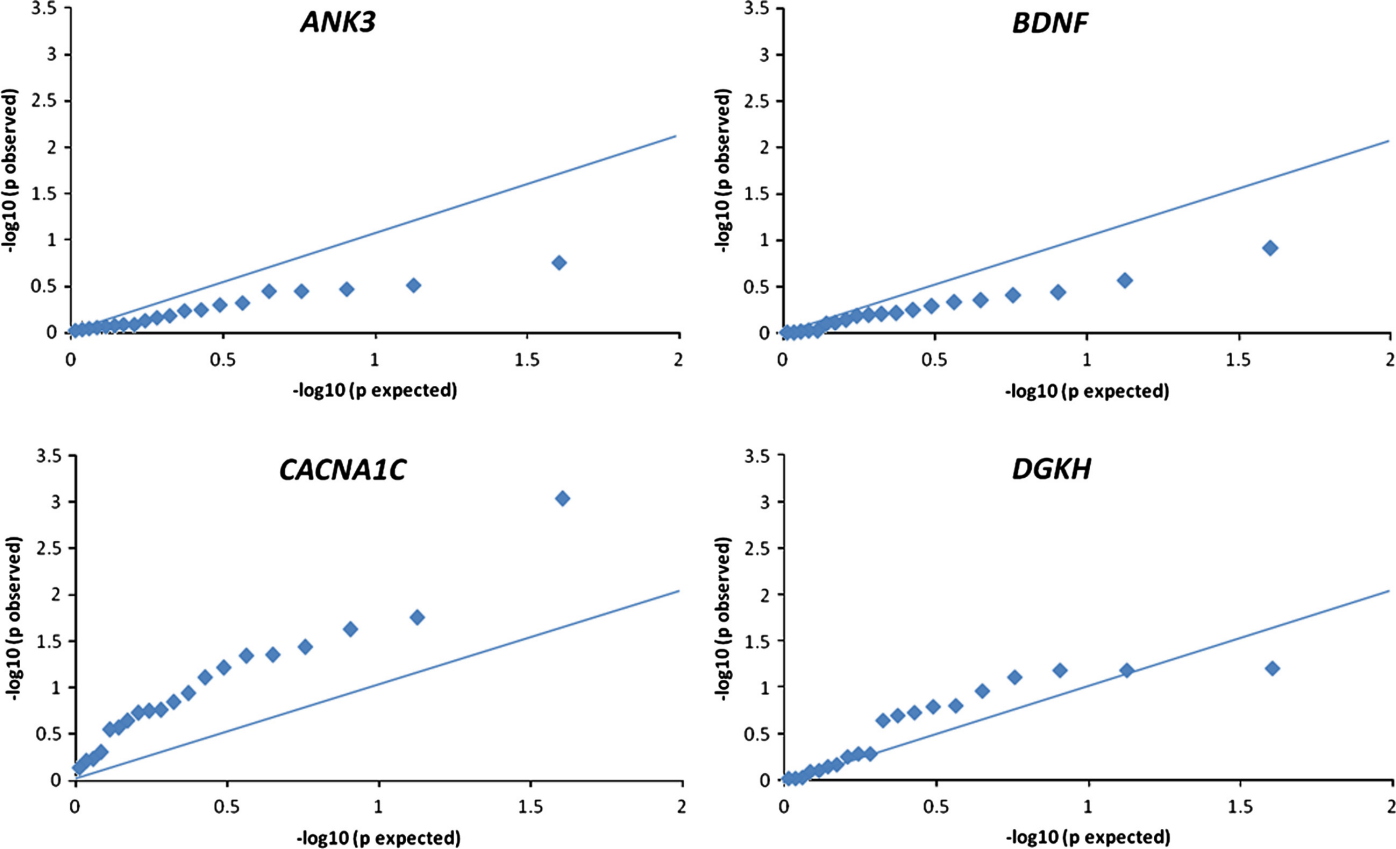
Q-Q plots of observed *P*-values by *P*-values expected under the null hypothesis for relationships between candidate genes and brain volumes. For *CACNA1C*, observed *P*-values are consistently more significant than those expected under the null hypothesis.

**Figure 2:**
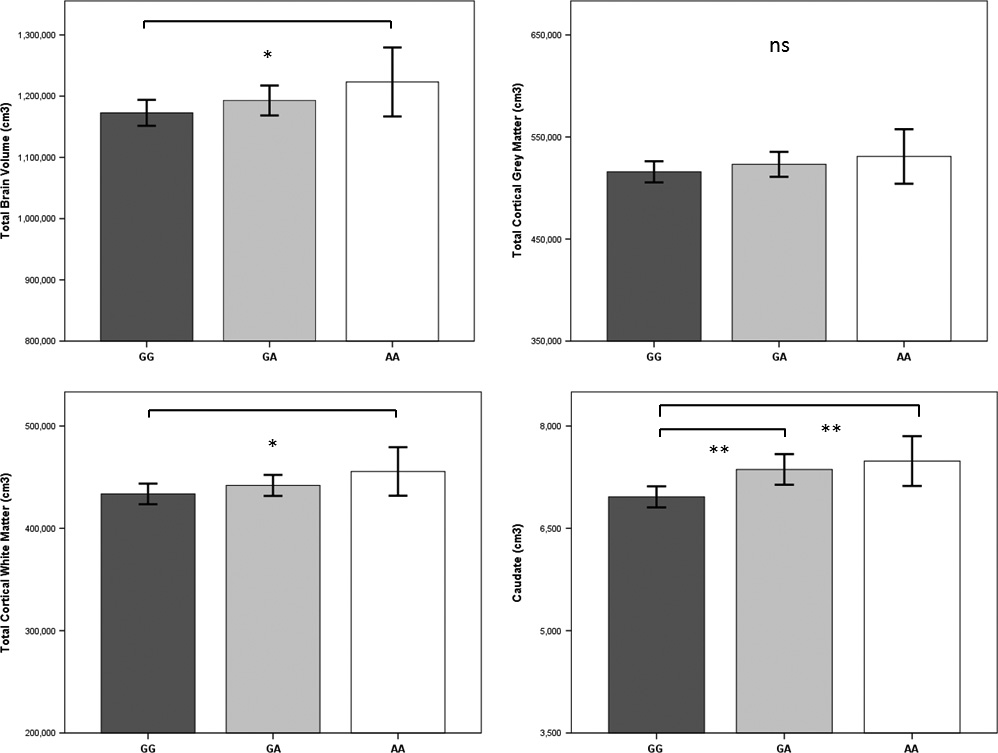
Total, cortical grey matter, cortical white matter, and caudate volumes by *CACNA1C* genotypes (GG *n* = 123, GA *n* = 103, AA = 33). Significant differences were observed between major allele homozygotes and minor allele carriers for total brain volume and total cortical white matter. For caudate volumes, minor allele genotype carriers differed significantly from the major allele homozygotes. *P < 0.05, **P < 0.001.

Table 4 presents relationships between candidate SNPs, total brain, and fronto-limbic volumes. *CACNA1C* minor allele carriers (GA/AA) showed significant positive relationships with total brain and total white matter volumes and fronto-limbic volumes in five of 17 regions examined, implying that the presence of minor alleles is related to greater brain volumes. Of these five significant fronto-limbic regions, only the caudate region remained significant following false discovery rate correction. Of the remaining 12 of 17 nonsignificant associations, 11 were in the positive direction, suggesting a general trend toward greater brain volumes with patients carrying at least one *CACNA1C* minor allele (sign test *P* < 0.0001). Figure 1 presents Q-Q plots of *P*-values from correlations between candidate SNPs and brain volumes. These plots indicate that relationships between *CACNA1C* minor allele carriers and brain volumes consistently deviated from the null hypothesis, suggesting a statistically reliable positive association with increased total brain and fronto-limbic volumes. Furthermore, the effect appears to be graded across homozygous minor, heterozygous, and major allele genotypes (Fig. 2). Covarying for race/ethnicity did not alter the pattern of *CACNA1C* clinical correlates. *ANK3, BDNF,* and *DGKH* genotypes were not significantly associated with total brain or fronto-limbic volumes in any region, with the exception of a negative relationship reflecting smaller anterior cingulate volume in *DGKH* minor allele carriers that did not survive false discovery correction. The pattern of relationships between genotypes, cognitive measures, and brain volumes remained the same after controlling for current psychotropic medication use and past history of substance use disorder.

**Table 4:** Relationships between candidate SNPs, total brain, and fronto-limbic volumes. Minor allele genotypes in *CACNA1C* were significantly associated with increased total brain volume and with volume of several fronto-limbic regions

	*ANK3 rs10994336 N* = 258	*BDNF rs6265 N* = 281	*CACNA1C rs1006737 N* = 259	*DGKH rs1170191 N* = 239
	*r*	*r*	*r*	*r*
Total brain volume	−0.06	0.00	0.13*	0.03
Total cortical grey matter	−0.01	0.03	0.08	−0.08
Total cortical white matter	−0.06	−0.04	0.13*	0.03
Frontal pole	−0.08	0.00	0.12*	−0.01
Superior frontal	−0.01	0.02	0.09	−0.07
Middle frontal	−0.04	0.00	0.07	−0.10
Lateral orbital frontal	0.01	−0.03	0.02	−0.09
Medial orbital frontal	−0.06	−0.05	0.08	−0.06
Anterior cingulate	−0.01	0.03	0.03	−0.13*
Nucleus accumbens	0.02	0.01	0.11	−0.07
Caudate	0.02	−0.02	0.21**	−0.05
Putamen	0.06	0.05	0.10	0.04
Globus pallidus	0.04	0.05	0.12*	0.04
Hippocampus	−0.04	0.04	0.04	0.01
Amygdala	0.01	−0.02	0.03	0.01
Insula	−0.03	0.09	0.14*	−0.09
Pars opercularis	0.03	−0.07	−0.08	−0.03
Pars triangularis	−0.03	0.00	0.08	−0.09
Pars orbitalis	−0.02	0.03	0.07	−0.02
Ventral diencephalon	0.01	0.03	0.15*	0.05

* *P* < 0.05,

** *P* < 0.001. Correlations were computed in all three study groups. The significant relationship between *CACNA1C* genotype groups and caudate volume survived false discovery rate correction. The relationship between *CACNA1C* genotype groups and caudate volume is significantly larger than the relationship between *CACNA1C* genotype groups and cortical grey matter volume [*t*(256) = 2.18, *P* = 0.030].

### Mediational models

**Figure 3:**
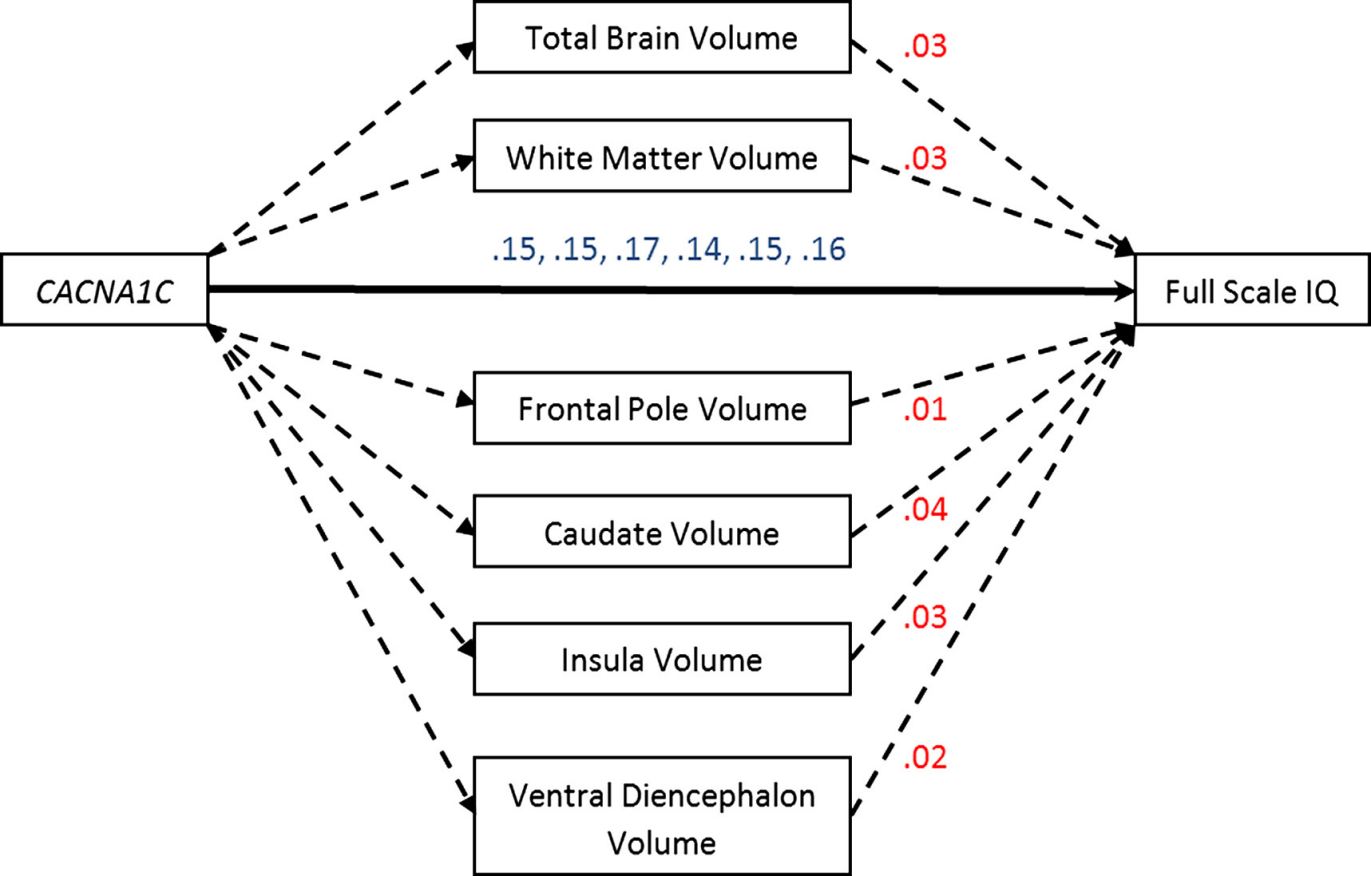
Mediational modeling results. *CACNA1C* minor allele genotype carriers had higher full scale IQ scores and this relationship was independent of increases in total and fronto-limbic brain volumes. Direct effects are given in blue (all *P* < 0.05) and listed in order from total brain to ventral diencephalon volume. Indirect effects are given in red (all *P* > 0.10).

The significant relationship between increased IQ and brain volumes with the patients carrying at least one *CACNA1C* minor allele raised the possibility that increased brain volumes may mediate increased IQ in individuals with these genotypes. Figure 3 presents mediational modeling results for the relationships between *CACNA1C* genotype group, brain volumes, and full scale IQ. *CACNA1C* minor allele genotypes increased full scale IQ scores independently of increases in total and fronto-limbic brain volumes. The same pattern of results was observed for verbal and nonverbal IQ scores. The nominally significant relationship between *DGKH* minor allele carriers and reduced verbal memory was not mediated by reduced anterior cingulate volumes.

## Discussion

The present data highlight the complexity of relationships between candidate genes, structural neural and cognitive endophenotypes, and mood disorder phenotypes.

None of the four genotypes tested showed significant association with categorical diagnoses (bipolar disorder, major depressive disorder, or any mood disorder), which is perhaps to be expected given the small sample size with regard to case/control association studies. Although not significant, it is interesting to note that the SNPs in the *BDNF* gene showed the strongest evidence of association with any mood disorder diagnosis, in comparison to specific diagnoses (bipolar disorder or major depressive disorder). This reinforces the notion that candidate polymorphisms may predispose to broader neural system dysfunction rather than to specific neural abnormalities that map to precise mood dysregulation patterns. Instead, the combination of numerous alleles may increase neural system vulnerability to mood dysregulation, and this vulnerability may then be further shaped by environmental influences and mood episode triggers.

The effects observed for *CACNA1C* further underscore the need to better understand the influence of candidate polymorphisms on neural system functioning and neuropsychiatric phenotypes. Previous large sample genetic association studies have supported a role of the *CACNA1C* minor allele in bipolar disorder and schizophrenia. Smaller sample studies of healthy and psychiatric disorder populations have found that the *CACNA1C* risk allele increases mRNA transcript (Bigos et al. 2010) and alters Akt pathway activation (Balog et al. 2010). These molecular changes result in increased brain volumes (Kempton et al. 2009; Wang et al. 2011), reductions in emotional and cognitive processing (Bigos et al. 2010; Krug et al. 2010), increased brain activation signals during cognitive processing (Bigos et al. 2010; Krug et al. 2010), and decreased regional connectivity (Wang et al. 2011). While the present study did not replicate associations between the *CACNA1C* polymorphism and mood disorder, this is likely due to the need for very large samples to detect weak associations between candidate polymorphisms and neuropsychiatric diagnoses (Ferreira et al. 2008; Sullivan et al. 2012). However, the present work does reinforce previous observations regarding stronger effects for the *CACNA1C* risk allele on cognitive and neuroimaging endophenotypes (Bigos et al. 2010) and clarifies the nature of these downstream phenotypic effects. *CACNA1C* minor allele carriers had increased global brain volume, with larger effects for specific fronto-limbic regions - especially the caudate. In contrast with previous literature (Kempton et al. 2009; Wang et al. 2011), total cortical white matter increases were a prominent driver of increased brain volume. Total cortical grey matter increases were present, but more modest and not statistically significant. The *CACNA1C* risk allele also appears to increase global cognitive ability. This is opposite of most findings suggesting reductions in specific aspects of cognitive and emotional processing (Bigos et al. 2010; Krug et al. 2010; Soeiro-de-Souza et al. 2012), but congruent with a recent study suggesting that *CACNA1C* minor allele carriers with bipolar disorder may have improved working memory (Zhang et al. 2012). Taken together, these data imply that *CACNA1C* risk allele effects may be valuable for some aspects of cognition, but harmful for others; an interpretation that fits with an evolutionary view of mood disorder (Akiskal and Akiskal 2005) and disease-associated genes as having both adaptive and nonadaptive value depending on context (Crespi et al. 2007; Tennessen and Akey 2011). Co-incident measurement of mood, global, and specific cognitive processes; brain structure and function; and downstream molecular mechanisms will be needed to more accurately characterize effects of this polymorphism.

The observation of stronger effects of the *CACNA1C* polymorphism on caudate volumes than on global brain volumes is intriguing. *CACNA1C* minor alleles have been associated with both bipolar disorder and schizophrenia and nonmotor caudate subregions have been found to be important for integrating cognitive and emotional processing. Thus, the *CACNA1C* polymorphism may preferentially predispose individuals toward mood disorder or schizoaffective phenotypes. An important next step will be to explore specificity in the patterns of emotion or cognitive dysfunction (developmental course, severity, episodes, etc.) in individuals with this genotype.

The present study is also the first to our knowledge to show that the strong positive relationship between *CACNA1C* minor allele genotypes and global cognitive ability was not mediated by total brain or fronto-limbic volume increases. The molecular mechanisms by which *CACNA1C* minor allele genotypes increase brain volumes, particularly fronto-limbic volumes, and independently increase IQ deserve further exploration. It is possible that increases in IQ are mediated by other brain regions not specifically investigated in the present study or that distinct molecular mechanisms influence neural system and cognitive vulnerability. Alternatively, *CACNA1C* influences on brain structure may be completely separate from influences on function. In this scenario, there may be distinct downstream molecular and cellular effects of the *CACNA1C* polymorphism that deserve elaboration.

*ANK3*, *BDNF*, and *DGKH* genotypes did not show significant bivariate relationships with imaging volumes or cognitive processes. However, there was a nominally significant relationship between *DGKH* minor allele genotypes and smaller anterior cingulate volumes and, independently, with reduced verbal memory. These observations are consistent with literature identifying a role of *DGKH* in mTOR and ERK1/2 signaling influencing cell growth (Merida et al. 2008), including regulation of neural morphology (dendritic branching and spine formation) in fronto-limbic regions and hippocampal long-term potentiation influencing memory (Shirai et al. 2010; Tu-Sekine and Raben 2011).

The major study limitations are the modest sample size for evaluating genetic associations with phenotypic characteristics (particularly within diagnostic groupings), restriction to fronto-limbic volumes, missing data on genotypes, lack of information on smoking status, and lack of ancestry-informative markers to provide more detailed evaluation of population stratification. In spite of the modest sample size for genetic associations with complex neuropsychiatric disease, this study is one of the largest cross-level (polymorphisms-structural imaging-cognition-diagnosis) studies completed to date. Multiple comparison corrections were applied at each stage of analysis to ensure that identified associations are not simply a result of multiple testing. Additionally, covarying for race/ethnicity did not alter the pattern of findings. However, future work should use ancestry-informative markers to more carefully examine population stratification issues.

The presence of missing data on genotypes may have influenced some of the relationships with phenotypic measures, although analyses suggest that any influence of missing data is likely minimal for the analyses presented. The focus on fronto-limbic volumes is both a strength and a weakness because it decreases concerns of Type I error that might arise from evaluation of all brain regions, but also may have resulting in missing important functional relationships. Additionally, lack of information on smoking status is unfortunate because smoking may influence brain volumes in psychiatric groups (Schneider et al. 2014). Future research examining genotype-phenotype relationships should examine both smoking and any other current or past substance abuse as these may be important moderating or confounding factors. Finally, the cross-sectional nature of the study is also a limitation, as longitudinal relationships between levels of the genotype-phenotype pathway and temporal sequencing of possible mediation could not be investigated. Future cross-level studies involving candidate genes would be wise to include multiple assessment points to clarify whether changes in molecular and neural systems measurements influence mood disorder progression. These studies can further elucidate whether molecular or neural system changes are influenced by genotype, as has been shown for BDNF and changes in brain volumes during recovery from drinking (Mon et al. 2013).

The present study greatly extends knowledge of the role of *CACNA1C* variation in brain structure, function, and vulnerability to mood dysregulation. It also provides a model, as well as a cautionary tale, that informs future cross-level studies evaluating the role of common genetic variation in complex neuropsychiatric diseases. Future large-scale multimodal studies will be needed to clarify relationships between candidate genes, structural and functional brain characteristics, and cognitive processes related to mood disorder vulnerability. These types of studies, if well-powered, have the potential to identify specific molecular-neural systems relationships involved in mood disorder. In doing so, translational studies may identify sensitive neural treatment targets for genetically informed therapeutics, enhancing the speed of development and efficacy evaluation of new medicines.
